# Estimating the potential value of MSM‐focused evidence‐based implementation interventions in three Ending the HIV Epidemic jurisdictions in the United States: a model‐based analysis

**DOI:** 10.1002/jia2.26265

**Published:** 2024-07-05

**Authors:** Benjamin Enns, Yi Sui, Brenda C. Guerra‐Alejos, Lia Humphrey, Micah Piske, Xiao Zang, Susanne Doblecki‐Lewis, Daniel J. Feaster, Victoria A. Frye, Elvin H. Geng, Albert Y. Liu, Brandon D. L. Marshall, Scott D. Rhodes, Patrick S. Sullivan, Bohdan Nosyk, the localized economic modelling study group

**Affiliations:** ^1^ Centre for Advancing Health Outcomes Vancouver British Columbia Canada; ^2^ School of Public Health University of Minnesota Minneapolis Minnesota USA; ^3^ Division of Infectious Diseases University of Miami Miller School of Medicine Miami Florida USA; ^4^ Department of Public Health Sciences University of Miami Miller School of Medicine Miami Florida USA; ^5^ School of Social Work Columbia University New York New York USA; ^6^ Center for Dissemination and Implementation Institute for Public Health Division of Infectious Diseases Department of Medicine School of Medicine Washington University in St. Louis St. Louis Missouri USA; ^7^ Bridge HIV San Francisco Department of Public Health San Francisco California USA; ^8^ Department of Epidemiology School of Public Health Brown University Providence Rhode Island USA; ^9^ Department of Social Sciences and Health Policy Wake Forest University School of Medicine Winston‐Salem North Carolina USA; ^10^ School of Public Health Emory University Atlanta Georgia USA; ^11^ Faculty of Health Sciences Simon Fraser University Burnaby British Columbia Canada

**Keywords:** HIV, implementation science, simulation modelling, cost‐effectiveness, economic evaluation, men who have sex with men

## Abstract

**Introduction:**

Improving the delivery of existing evidence‐based interventions to prevent and diagnose HIV is key to Ending the HIV Epidemic in the United States. Structural barriers in the access and delivery of related health services require municipal or state‐level policy changes; however, suboptimal implementation can be addressed directly through interventions designed to improve the reach, effectiveness, adoption or maintenance of available interventions. Our objective was to estimate the cost‐effectiveness and potential epidemiological impact of six real‐world implementation interventions designed to address these barriers and increase the scale of delivery of interventions for HIV testing and pre‐exposure prophylaxis (PrEP) in three US metropolitan areas.

**Methods:**

We used a dynamic HIV transmission model calibrated to replicate HIV microepidemics in Atlanta, Los Angeles (LA) and Miami. We identified six implementation interventions designed to improve HIV testing uptake (“Academic detailing for HIV testing,” “CyBER/testing,” “All About Me”) and PrEP uptake/persistence (“Project SLIP,” “PrEPmate,” “PrEP patient navigation”). Our comparator scenario reflected a scale‐up of interventions with no additional efforts to mitigate implementation and structural barriers. We accounted for potential heterogeneity in population‐level effectiveness across jurisdictions. We sustained implementation interventions over a 10‐year period and evaluated HIV acquisitions averted, costs, quality‐adjusted life years and incremental cost‐effectiveness ratios over a 20‐year time horizon (2023–2042).

**Results:**

Across jurisdictions, implementation interventions to improve the scale of HIV testing were most cost‐effective in Atlanta and LA (CyBER/testing cost‐saving and All About Me cost‐effective), while interventions for PrEP were most cost‐effective in Miami (two of three were cost‐saving). We estimated that the most impactful HIV testing intervention, CyBER/testing, was projected to avert 111 (95% credible interval: 110–111), 230 (228–233) and 101 (101–103) acquisitions over 20 years in Atlanta, LA and Miami, respectively. The most impactful implementation intervention to improve PrEP engagement, PrEPmate, averted an estimated 936 (929–943), 860 (853–867) and 2152 (2127–2178) acquisitions over 20 years, in Atlanta, LA and Miami, respectively.

**Conclusions:**

Our results highlight the potential impact of interventions to enhance the implementation of existing evidence‐based interventions for the prevention and diagnosis of HIV.

## 1 INTRODUCTION

In 2019, the Ending the HIV Epidemic (EHE) initiative was announced in the United States, with the goal of reducing HIV incidence by 90% in priority jurisdictions by 2030 [1, 2]. Despite consistent increases in annual funding since its inception, however, the initiative is expected to fall short of these ambitious goals [3]. Prior work examining the population‐level scale‐up of HIV interventions across six cities estimated that overall expenditures would peak in 2024 if scale‐up began in 2020 [4]. COVID‐19 lockdowns resulted in service disruptions, compounding already suboptimal HIV testing rates [5, 6, 7], and reducing the use of pre‐exposure prophylaxis (PrEP) in some settings [5, 6, 8]. Furthermore, initial gains from evidence‐based biomedical interventions to prevent, diagnose and treat HIV have slowed, as efforts to expand uptake face both structural and implementation barriers [9, 10]. Implementation science offers a systematic and multidisciplinary approach to study the delivery of public health interventions [11, 12], which applies established frameworks to optimize the scalability of innovations while identifying and reducing barriers to implementation [13, 14, 15]. In order to advance the HIV response through implementation science, studies must capture the potential heterogeneity of interventions across settings [16].

Chaudoir et al. proposed a multi‐level framework for evaluating key factors affecting the successful implementation of evidence‐based interventions (i.e. those that provide the strongest evidence of efficacy for PrEP, HIV risk, medication adherence and engagement in HIV care) including structural factors at the top level, with organizational‐, provider‐, patient‐ and innovation‐level factors below (e.g. quality of evidence and complexity of the intervention) [17, 18, 19]. Structural barriers entail challenges which require larger‐scale policy interventions to overcome (e.g. drug prices, insurance coverage and access to health services). Implementation barriers arise when biomedical interventions are available, but uptake is hindered by stigma, awareness, deficits in health literacy or challenges in integration into routine clinical practice. In the case of public health interventions for HIV, structural barriers such as underinsurance are often decisive factors in client decision‐making. Structural barriers for PrEP uptake differ across jurisdictions, with drug costs and insurance coverage being prominent factors [20, 21, 22]. PrEP‐to‐need ratios are lowest in Southern states compared to national estimates (8.8 vs. 12.1), which have higher proportions of uninsured, Latinx or Black populations, and states without Medicaid expansion [23, 24, 25, 26].

Independent of structural constraints, however, EHE efforts are challenged by structural racism, stigma and poor socio‐economic conditions, particularly among Black and Hispanic/Latinx men who have sex with men (MSM) [27, 28, 29]. Implementation interventions may help to reduce racial and ethnic disparities in access, and there are several examples of successful implementation interventions in the United States, including geographically focused HIV screening in underserved areas of Philadelphia [30], and pharmacist‐led PrEP in multiple states [31, 32, 33]; however, evidence of uptake and expansion of these interventions beyond their initial trials is limited. Recent studies have synthesized the existing evidence base for interventions focused on both PrEP [34] and HIV testing [35], as well as the costs of implementation interventions [36]. With this evidence, simulation model‐based cost‐effectiveness analysis (CEA) can help refine implementation interventions, and promote further expansion by demonstrating their long‐term impact and value [37].

Our objective was to estimate the potential value and epidemiological impact of implementation interventions to improve existing HIV testing and PrEP interventions. We proceed with illustrative case studies in three US jurisdictions, to estimate the potential value of improved implementation, and highlight the need for better implementation data to support public health efforts.

## 2 METHODS

### 2.1 Localized economic modelling of HIV microepidemics

We adapted and calibrated a dynamic, compartmental HIV transmission model to replicate HIV microepidemics in Atlanta, Los Angeles (LA) and Miami, which encompass six EHE focal counties. We selected these cities, as they represent unique and disparate implementation contexts, including different demographic composition, differences in Affordable Care Act (ACA) adoption, as well as the size and scope of HIV epidemic [38]. The model tracked individuals susceptible to HIV acquisition through the course of infection, diagnosis, treatment with antiretroviral therapy (ART) and ART dropout. In each city, we stratified the adult population (age 15–64 years) by sex at birth, HIV risk group (MSM, people who inject drugs, MSM who inject drugs and heterosexuals), racial/ethnic group (Black/African American, Hispanic/Latinx and non‐Hispanic/Latinx White/others) and sexual risk behaviour (high‐ vs. low‐risk). Details of the evidence synthesis [39], model development and calibration [40], interventions [9], and CEA of combination implementation strategies [41] have been published separately.

### 2.2 Modelling intervention scale‐up using the RE‐AIM framework

We estimated the potential population‐level scale and effectiveness of opt‐out HIV testing, and PrEP among MSM in primary care settings using the Reach Effectiveness Adoption Implementation Maintenance (RE‐AIM) framework [42], defining these components, along with costs of implementation, delivery and sustainment of each intervention (Table S1) [9]. In the model, testing effectiveness was defined as the population‐level testing rate in each jurisdiction (stratified by racial/ethnic group and HIV risk group), and PrEP effectiveness was defined as PrEP efficacy at optimal dosing combined with the proportion of the population adherent to optimal dosing [39]. Status quo testing rates were estimated via model calibration [40], while PrEP coverage was estimated from surveillance data [39]. Scale of delivery was the product of reach and adoption, with reach defined as the participation rate in a given intervention in the target population, conditional on both the probability an individual would access services and would accept the intervention, and adoption defined as the proportion of delivery settings implementing the intervention [9, 42]. Our baseline of comparison was interventions delivered at a previously documented scale [9].

### 2.3 Modelling the effect of implementation interventions

We specified implementation interventions as strategies that target improvements to RE‐AIM components of biomedical interventions, to increase their scale beyond the levels estimated in Krebs et al. [9]. We selected two existing biomedical interventions: opt‐out HIV testing in primary care, and PrEP for indicated MSM. Full‐scale implementation of a biomedical intervention would entail reaching the entire target population, whereas the actual level of implementation reflects the combination of structural and implementation barriers preventing some of the target population from accessing an intervention. Structural barriers included laws and policies (e.g. HIV criminalization laws), geographic location and financial impediments (e.g. insurance coverage) which inhibited the uptake of HIV interventions, that cannot be addressed through interventions at the local public health level [10, 43]. Thus, we assumed that the maximum potential impact of implementation interventions was limited by the ceiling imposed by structural barriers.

We identified implementation interventions for HIV testing and PrEP from two recent systematic reviews by Wang et al. (Table 1) [34, 35]. We selected HIV testing and PrEP interventions that specifically focused on improving the reach, adoption or effectiveness of existing HIV interventions, and modelled the increased scale or effectiveness that could be achieved by the addition of these interventions (Table 2). Our comparator scenario was based on previously documented scales of delivery for biomedical interventions [9], which reflected population characteristics, as well as structural and implementation barriers at the jurisdiction or regional level. We assumed that the efficacy of implementation interventions would be as reported in the source studies; however, we also assumed that the population‐level effectiveness of each implementation intervention may not be replicated outside of the trial setting. We specified limits according to structural constraints (i.e. estimated insurance coverage based on state ACA adoption status), as well as constraints in uptake specific to each intervention (Table S2). Our methods for deriving implementation intervention costs are detailed in the Supplement. We also consulted with the authors of the source studies to verify cost and scale estimates (Table S10).

**Table 1:** Description of implementation interventions to improve delivery of opt‐out HIV testing in primary care settings and PrEP among MSM

Implementation intervention	Description	Study location (size)	Study setting	Target population	Study design	Trial effectiveness
**Biomedical intervention: Opt‐out HIV testing in primary care settings**						
Academic detailing for HIV testing	HIV needs/knowledge assessment and 1‐hour training sessions for non‐HIV specialty healthcare providers to increase routine HIV testing in specialty care settings.	Chicago, IL (*N* = 43)	Non‐HIV specialty clinics	MSM	Pre‐Post	30% increase (proportion tested at baseline vs. post‐intervention; average among all settings)
CyBER/testing	Online content on MSM‐frequented social media hosted by a health educator whose profile includes HIV testing‐related content and local resources; able to ask private questions.	USA (*N* = 1292)	Online social/sexual networks	MSM	RCT	61% increase (risk‐ratio approximation from baseline 42% [probability of testing in control group] and 2.9 AOR)
All About Me	Personalized computer‐based HIV testing approach recommendation.	New York, NY (*N* = 236)	General population	Young Black MSM	RCT	36% increase (average of intervention and control groups)
**Biomedical intervention: PrEP among indicated MSM**						
Project SLIP	Eligibility screening questionnaire and linkage in primary care settings.	Southern California (*N* = 36)	Primary care	MSM	Pre‐Post	22% increase in PrEP referrals (22% of referrals during intervention period attributed to screener)
PrEPmate	Interactive text‐messaging check‐ins and reminders for PrEP retention and adherence for young MSM.	Chicago, IL (*N* = 121)	Public health clinic	Young MSM	RCT	28% increase in adherence (risk‐ratio approximation from baseline [57% in control group] and 2.06 AOR)
PrEP patient navigation	Strengths‐based case management with patient navigators to promote and facilitate linkage to a PrEP‐competent provider and PrEP uptake.	Southern Florida (*N* = 61)	Community‐based	HIV‐negative MSM	RCT	21% (difference in PrEP initiation between treatment and control groups at 12 weeks)

Abbreviations: AOR, adjusted odds‐ratio; CyBER, Cyber‐Based Education and Referral; HIV, human immunodeficiency virus; IL, Illinois; MSM, men who have sex with men; NY, New York; PrEP, pre‐exposure prophylaxis; RCT, randomized controlled trial; SLIP, Screening and Linkage Intervention in Primary Care; USA, United States of America.

**Table 2:** Improvements to existing HIV testing and PrEP biomedical interventions resulting from implementation interventions

Implementation intervention	Target RE‐AIM component	Trial‐based effectiveness	Model population	Total scaling factor^a^	Population‐level effect
**Biomedical intervention: Opt‐out HIV testing in primary care settings**					
Academic detailing for HIV testing	Adoption	30% increase (proportion tested at baseline vs. post‐intervention; average among all settings)	MSM	Combined scale limit: 16% (ATL and MIA); 19% (LA)	ATL and MIA: 5% LA: 6%
CyBER/testing	Reach	61% increase (risk‐ratio approximation from baseline 42% [probability of testing in control group] and 2.9 AOR)	MSM	Combined scale limit: 30% (ATL and MIA); 37% (LA)	ATL and MIA: 19% LA: 23%
All About Me	Reach	68% increase (weighted average overall increase in testing at 6 months across both groups vs. baseline [72% vs. 43%])	MSM (Black; aged 16–29)	Combined scale limit: 12% (ATL and MIA); 15% (LA)	ATL and MIA: 8% LA: 10%
**Biomedical intervention: PrEP among indicated MSM**					
Project SLIP	Adoption	17% increase in individuals on PrEP (22% [increase in PrEP referrals] multiplied by 77% [filled PrEP prescriptions among referrals])	MSM (indicated for PrEP)	Combined scale limit: 8% (ATL and MIA); 10% (LA)	ATL and MIA: 1% LA: 2%
PrEPmate	Effectiveness	28% increase in effectiveness (improved adherence risk‐ratio approximation from baseline [57% in control group] and 2.06 AOR)	MSM (indicated for PrEP)	Scale limit (Effectiveness): 91% (All cities)	(Effectiveness) All cities: 26%
PrEP patient navigation	Reach	21% (difference in PrEP initiation between treatment and control groups at 12 weeks)	MSM (indicated for PrEP)	Combined scale limit: 39% (ATL and MIA); 48% (LA)	ATL and MIA: 8% LA: 10%

Abbreviations: AOR, adjusted odds‐ratio; ATL, Atlanta; CyBER, Cyber‐Based Education and Referral; HIV, human immunodeficiency virus; LA, Los Angeles; MIA, Miami; MSM, men who have sex with men; PrEP, pre‐exposure prophylaxis; RE‐AIM, reach, effectiveness, adoption, implementation, maintenance; SLIP, Screening and Linkage Intervention in Primary Care.

^a^ Scaling factor represents the estimated limitation to population‐level scale (relative to full scale of 100%) as a result of intervention‐specific and structural constraints.

### 2.4 Implementation interventions for HIV testing

For HIV testing, we selected an academic detailing intervention to increase HIV testing offers at non‐speciality clinics, hoping to capture a wider demographic of patients who may not have sought out HIV care otherwise (“Academic detailing for HIV testing”) [44]. We also selected two patient‐focused interventions involving testing information and interactive educational support offered online through sexual networking social media and web platforms to target MSM (“CyBER/testing”) [45], as well as personalized computer‐based HIV testing recommendations for young Black MSM (“All About Me”) [46].

### 2.5 Implementation interventions for PrEP

We selected two interventions focused on improving patient‐level factors (increased reach and effectiveness via PrEP adherence) by providing support in the form of interactive text message reminders (“PrEPmate”) [47] and strengths‐based case management (“PrEP patient navigation”) [48] offered by trained healthcare professionals and navigators for MSM initiating PrEP. Additionally, we selected one intervention which offered provider‐side improvements (increased adoption) in the form of educational training and in‐office PrEP screening questionnaires to help physicians integrate PrEP consultation and referral into visits from eligible candidates (“Project SLIP”) [49]. Given that regular HIV testing is a mandatory component of PrEP, we handled this separately from general HIV testing interventions, which were only applied to those who were susceptible or undiagnosed and not on PrEP.

### 2.6 Model outcomes and CEA

We estimated quality‐adjusted life‐years (QALYs), total costs (including implementation intervention costs), as well as other costs such as medication and healthcare utilization, in 2022 (USD). We reported incident HIV acquisitions averted, as this is an important metric of the EHE initiative, and for comparability with other modelling studies, however, incidence was a secondary outcome in this analysis. We sustained the estimated effects of the interventions over a 10‐year period, with a 20‐year time horizon (2023–2042) for evaluating outcomes (to capture second‐order effects of reduced HIV transmission). We assumed a scale‐up period of 18 months to reach the target scale of delivery, followed by a sustainment period, where interventions were held at the target level of scale. We estimated incremental cost‐effectiveness ratios (ICERs) as the incremental cost per QALY gained for each implementation intervention, relative to the comparator scenario. We assumed the additional costs related to interventions would be fully funded and conducted our analysis from a healthcare sector perspective, with costs and QALYs discounted at 3% annually. We evaluated ICERs against a threshold of $100,000/QALY, which is commonly used and has been recommended as a threshold in the United States [50, 51].

### 2.7 Sensitivity analysis

Our primary results were derived from the average of 2000 probabilistic sensitivity analysis simulations, presented as the percentage of simulations in which the intervention was cost‐effective according to the stated ICER threshold. Given the uncertainty in costs of implementation intervention scale‐up and delivery, we also conducted a deterministic threshold sensitivity analysis on the costs of each implementation intervention in each jurisdiction to determine the level of increased/decreased costs at which the cost‐effectiveness decision would change.

### 2.8 Ethical considerations

The study received ethics approval by the Simon Fraser University Office of Research Ethics and the University of British Columbia/Providence Health Care's Research Ethics Board (H16‐00652). As this study involved analysis of aggregate‐level and publicly available data, informed consent was not applicable.

## 3 RESULTS

### 3.1 Implementation interventions to improve HIV testing uptake

**Figure 1:**
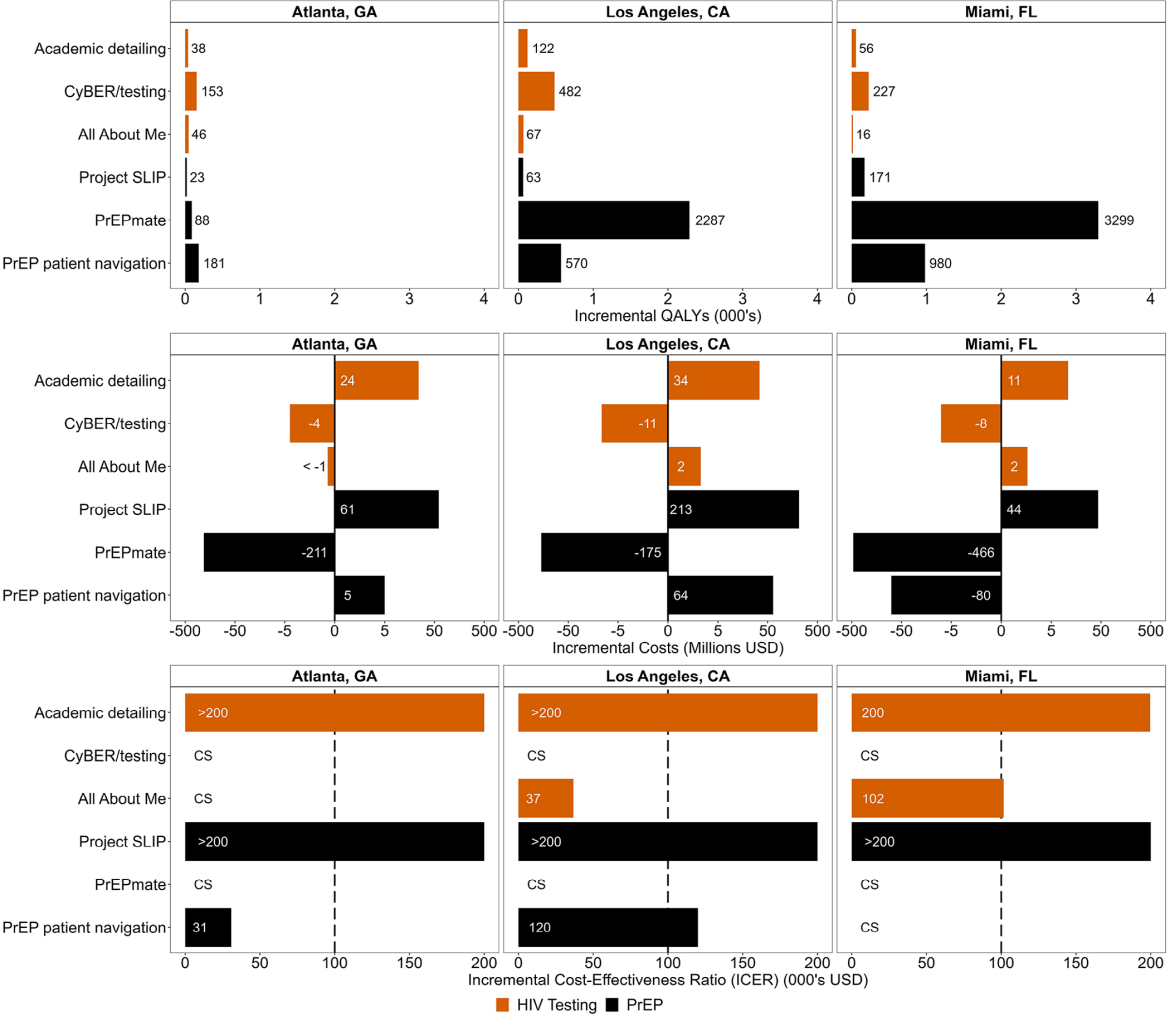
**Cost‐effectiveness outcomes over 20‐year time horizon for HIV testing and PrEP implementation interventions in Atlanta, LA and Miami**. Abbreviations: CA, California; CyBER, Cyber‐Based Education and Referral; FL, Florida; GA, Georgia; HIV, human immunodeficiency virus; ICER, incremental cost‐effectiveness ratio; LA, Los Angeles; PrEP, pre‐exposure prophylaxis; QALY, quality‐adjusted life year; SLIP, Screening and Linkage Intervention in Primary Care; USD, United States dollar.

**Figure 2:**
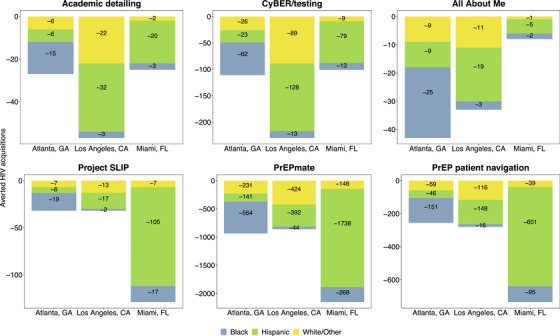
**Cumulative HIV acquisitions averted by HIV testing and PrEP implementation interventions for Atlanta, LA and Miami over a 20‐year time horizon**. Abbreviations: CA, California; CyBER, Cyber‐Based Education and Referral; FL, Florida; GA, Georgia; HIV, human immunodeficiency virus; LA, Los Angeles; PrEP, pre‐exposure prophylaxis; SLIP, Screening and Linkage Intervention in Primary Care.

Academic detailing for HIV testing was not projected to be cost‐effective in any jurisdiction (ICERs: ≥$200,000/QALY; <1% of simulations indicated the intervention was cost‐effective across cities) (Figure 1), and we estimated that it would avert an additional 27 (95% credible interval: 27–28), 57 (57–59) and 25 (25–25) HIV acquisitions in Atlanta, LA and Miami, respectively (Figure 2). CyBER/testing was projected to be cost‐saving in all jurisdictions, and we estimated that it would avert an additional 111 (110–111), 230 (228–233) and 101 (101–103) HIV acquisitions in Atlanta, LA and Miami, respectively. All About Me was projected to be cost‐saving in Atlanta, cost‐effective in LA and not cost‐effective in Miami (ICER: $37,000/QALY; 98% cost‐effective, and $102,000/QALY; 53% cost‐effective). We estimated that All About Me would avert 43 (43–43), 33 (33–33) and 8 (8–8) HIV acquisitions in Atlanta, LA and Miami, respectively. Most HIV cases averted by implementation interventions for HIV testing were among Hispanic/Latinx MSM in Miami (63–80% across interventions), and most of the cases averted in Atlanta were among Black MSM (56–58% across interventions). As a result of the focus on Black MSM, All About Me had the largest impact of any intervention in Atlanta, which differed from other testing interventions where the impact in Atlanta was smaller. This also resulted in a smaller impact in LA and Miami, where the majority of new HIV cases are among Hispanic/Latinx MSM.

### 3.2 Implementation interventions to improve PrEP uptake

Project SLIP was not projected to be cost‐effective in any jurisdiction (ICERs: >$200,000/QALY; <1% cost‐effective across cities) (Figure 1), and we estimated that it would avert an additional 32 (32–32), 32 (31–32) and 129 (126–131) HIV acquisitions in Atlanta, LA and Miami, respectively, over the 20‐year study time horizon (Figure 2). PrEPmate was projected to be cost‐saving in all jurisdictions, and we estimated that it would avert an additional 936 (929–943), 860 (853–867) and 2152 (2127–2178) HIV acquisitions in Atlanta, LA and Miami, respectively. PrEP patient navigation was projected to be cost‐saving in Miami, cost‐effective in Atlanta (ICER: $31,000/QALY; 97% cost‐effective), but above the cost‐effectiveness threshold in LA (ICER: $120,000/QALY; 34% cost‐effective). We estimated that PrEP patient navigation would avert 256 (255–258), 280 (277–283) and 735 (722–747) HIV acquisitions in Atlanta, LA and Miami, respectively. The overwhelming majority of HIV cases averted by PrEP implementation interventions were among Hispanic/Latinx MSM in Miami (81–82% across interventions), and most of the cases averted in Atlanta were among Black MSM (59–60% across interventions). PrEP implementation interventions were not specifically targeted by racial/ethnic group, therefore, results reflected the underlying epidemiological conditions in each jurisdiction, where most new cases in Atlanta were among Black MSM, and among Hispanic/Latinx MSM in Miami.

### 3.3 Threshold sensitivity analysis

**Figure 3:**
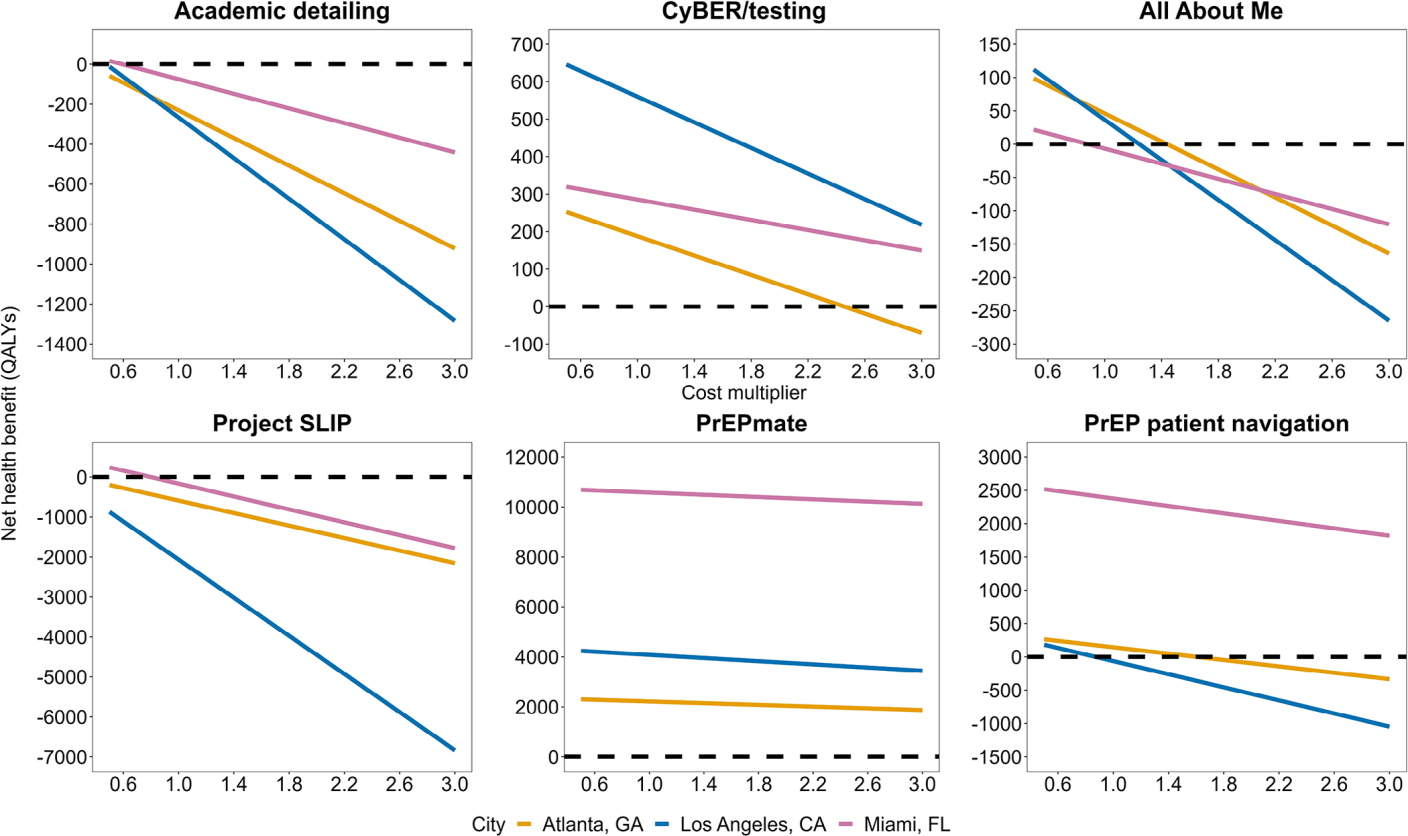
**Results of threshold sensitivity analysis on implementation intervention costs**. Abbreviations: CA, California; CyBER, Cyber‐Based Education and Referral; FL, Florida; GA, Georgia; PrEP, pre‐exposure prophylaxis; QALY, quality‐adjusted life year; SLIP, Screening and Linkage Intervention in Primary Care. Net health benefit is calculated relative to the cost‐effectiveness threshold, and defined as incremental QALYs minus health opportunity costs (incremental costs divided by the cost‐effectiveness threshold of $100,000 per QALY). All interventions produced positive health gains overall.

In threshold sensitivity analysis, we estimated that CyBER/testing would remain cost‐effective at $100,000/QALY at >3x increased costs in LA and Miami; however, in Atlanta, it would not be cost‐effective beyond a 2.5x increase (Figure 3). All About Me would remain cost‐effective in Atlanta and LA at moderately increased costs (20–50% increase), but was slightly above the cost‐effectiveness threshold in Miami, so any decrease in costs would change the cost‐effectiveness decision at $100,000/QALY. Academic detailing for HIV testing was estimated to be cost‐effective in Miami at a 40% reduction in base costs, however, would require further reductions (or improvements in population‐level effectiveness) in Atlanta and LA to become cost‐effective. We found that PrEPmate would be cost‐effective at >3x increased costs across jurisdictions, PrEP patient navigation would remain cost‐effective at $100,000/QALY in Miami at >3x increased costs, however, would no longer be cost‐effective in Atlanta and LA beyond 1.4x to 1.8x increased costs. Project SLIP was estimated to be cost‐effective in Miami at a 40% reduction in base costs, but would require further reductions in Atlanta, and LA (>50% reduction) before becoming cost‐effective at $100,000/QALY.

## 4 DISCUSSION

In this study, we examined the potential public health value and epidemiological impact of implementation interventions to improve opt‐out HIV testing in primary care, and PrEP for MSM. Implementation interventions for PrEP resulted in larger overall population health gains compared to HIV testing interventions in Miami, while in Atlanta, population health improvements were comparable across interventions. PrEPmate and CyBER/testing were cost‐saving across all jurisdictions, while academic detailing implementation interventions (Project SLIP and Academic detailing for HIV testing) were not projected to be cost‐effective in any jurisdiction at base costs. Implementation interventions for PrEP were most cost‐effective in Miami, while implementation interventions for opt‐out HIV testing in primary care were most cost‐effective in Atlanta. None of the implementation interventions generated large enough incidence reductions to approach 2030 EHE targets. While reductions in incidence are an important metric in evaluating progress towards EHE, it was not our goal to estimate whether these interventions in isolation could reach EHE targets. Prior studies have estimated that even combinations of interventions across all EHE pillars would only be able to achieve a maximum 50% reduction in incidence by 2030 [41]. Our findings instead provide a value‐based argument for the inclusion of implementation interventions as part of an ensemble of interventions in the EHE plans for local jurisdictions.

Our results reflected the underlying epidemiological conditions of each setting, where heterogeneous demography and large disparities in insurance coverage, healthcare access and employment are present [38, 52]. Notably, All About Me, designed specifically for young, Black MSM, was most cost‐effective in Atlanta given the high incidence rates among Black MSM, as well as racially segregated sexual networks [53]. A similar targeted focus on Hispanic/Latinx MSM could potentially make this type of intervention more cost‐effective in Miami. Out of the 42 interventions for HIV testing and PrEP uptake and adherence included in the systematic reviews by Wang et al. [34, 35], approximately 30% focused on Black or Hispanic/Latinx MSM. A modelling study of PrEP allocation strategies in LA county found allocating PrEP resources preferentially to Black individuals would have a substantial positive impact on increasing equity, with disparities in HIV incidence substantially reduced [54]. While HIV incidence may already be at parity between Black and White populations in some US settings [55], differences are significant in the South, where Black individuals account for more of the overall population than other parts of the country (e.g. 31% in Georgia vs. 13% nationally) [52, 56]. These inequities, largely driven by structural barriers, long‐standing social injustices and racism [10, 52, 57, 58], require interventions that address and bridge these disparities.

Structural barriers attenuated the impact of all interventions we evaluated. The overall scale of implementation interventions (with the exception of PrEPmate) was limited by the estimated proportion of MSM in the South and West regions with any health insurance [59], and would be lower if only considering those who were fully insured. Among participants in a demonstration project that provided PrEP free of charge in a research environment [60], racial/ethnic and sexual minority (i.e. MSM and transgender women) participants located in Miami were less likely to utilize PrEP beyond completion of a demonstration project study compared to those in San Francisco [61]. This highlights the critical role that insurance access plays in limiting the potential scale of PrEP interventions [20, 62, 63]. As health insurance coverage continues to be a substantial barrier in limiting the potential scale of PrEP, Medicaid expansion and PrEP Drug Assistance Programs improve equity in access to care in addition to policies that increase PrEP coverage and reduce related costs in insurance plans, and interventions which offer PrEP at no cost regardless of insurance coverage or immigration status [22, 23, 64, 65].

### 4.1 Limitations

This study had several limitations. First, we did not account for behavioural changes or service disruptions resulting from the COVID‐19 pandemic (e.g. reduced testing and treatment initiation, fewer sexual contacts); however, evidence has shown that HIV‐related service disruptions had largely resolved after a year [66]. Second, data to characterize the impact and costs of implementation interventions delivered at a population‐level were scarce, highlighting a need to document this in any future population‐level scale‐up efforts. To address this, we collaborated with the authors of the source studies to verify our modelling assumptions for their specific intervention (one or more authors from four of six studies agreed to participate in this work). We used conservative estimates when extrapolating trial‐based effectiveness estimates at a population level to avoid overly optimistic cost‐effectiveness estimates of implementation interventions for different jurisdictions. This also resulted in small credible intervals around estimates of averted HIV acquisitions, due to the relatively small population‐level impact in some jurisdictions (e.g. <50 averted over 20 years). Given these concerns, we conducted a threshold sensitivity analysis to determine the magnitude of increased (or decreased) costs at which the cost‐effectiveness decision was changed. Despite small population‐level impacts on incidence in some cases, many of these interventions remained cost‐effective with large cost increases. Third, we only applied implementation interventions for HIV testing to opt‐out testing in primary care in this analysis; however, CyBER/testing and All About Me were mobile health interventions that could also be applied to different testing modalities (e.g. self‐testing or non‐clinic‐based testing). Thus, our results may understate the potential impact of these interventions. Given that this analysis was intended to provide an illustrative case‐study on the potential value of implementation interventions, our selection of interventions was not comprehensive. Finally, the limitations in the underlying evidence base, calibration and model structure have been described in detail elsewhere [9, 39, 40]. Despite these challenges, we believe this work represents the first attempt to estimate the potential impact and cost‐effectiveness of HIV implementation interventions, while accounting for local heterogeneity.

## 5 CONCLUSIONS

Our results highlight the impact of interventions to enhance the delivery of existing evidence‐based interventions for the prevention and diagnosis of HIV. While total incidence reductions were relatively modest, the majority of interventions would be cost‐effective, and four were cost‐saving in at least one jurisdiction. Though funding for such interventions through public health agencies or other payors will necessarily differ across jurisdictions, subject to financial and human resource constraints, this study demonstrates such investments would provide good value for money to the broader healthcare system, according to commonly recommended thresholds. Cost‐effectiveness depended largely on implementation and epidemiological context—both the extent to which existing interventions are delivered sub‐optimally (e.g. imperfect PrEP adherence, or low acceptance of routine HIV testing), and the potential for improvements tailored to the local epidemic, such as a focus on Black MSM. Our findings suggest that there is value in improving the implementation and delivery of existing HIV interventions; however, jurisdictions should identify these important characteristics to target implementation interventions most effectively. While structural barriers to access are critical, and require higher‐level policy interventions, there should be greater emphasis on improving implementation to support the goals of the EHE initiative.

## COMPETING INTERESTS

The authors have no competing interests to declare.

## AUTHORS’ CONTRIBUTIONS

BE, LH, YS, XZ and BN designed the study. BE wrote the first draft of the article. BE, LH and BCG‐A conducted the evidence synthesis. LH, YS and XZ executed the modelling analysis. BE, BCG‐A, YS and MP wrote the final draft of the article. AYL, DJF, SD‐L, SDR and VAF provided consultation on key model parameters for the source interventions. AYL, BDLM, DJF, EHG, PSS, SD‐L, SDR, VAF and BN aided in the interpretation of results and provided critical revisions to the article. BN secured funding for the study. All authors approved the final draft.

## FUNDING

This study was supported by the National Institutes on Drug Abuse (R01DA041747; PI: Nosyk). The funding agreement ensured the authors’ independence in designing the study, interpreting the data, writing and publishing the report.

## CME STATEMENT

This article is published as part of a supplement supported by unrestricted educational grant by ViiV Healthcare.

Credits Available for this Activity: American Medical Association (AMA Credit).

Washington University School of Medicine in St. Louis designates this enduring material for a maximum of 1 AMA PRA Category 1 Credit™. Physicians should claim only the credit commensurate with the extent of their participation in the activity.

## Supporting information


**Additional file 1**: Contains supplementary information on intervention effect parameterization, estimation of intervention costs, and Tables S1 to S10 and Figure S1

## Data Availability

The data that support the findings of this study are available in the Supporting Information of this article or in published literature.
