# Supplementary material for: Estimating the potential value of MSM‐focused evidence‐based implementation interventions in three Ending the HIV Epidemic jurisdictions in the United States: a model‐based analysis
Source: J Int AIDS Soc. 2024 Jul 5;27(Suppl 1):e26265. doi: 10.1002/jia2.26265 (PMC11224592; doi:10.1002/jia2.26265)
Supplement: Supplementary file 1 — Additional file 1: Contains supplementary information on intervention effect parameterization, estimation of intervention costs, and Tables S1 to S10 and Figure S1 [file JIA2-27-e26265-s001.docx]

**SUPPLEMENTAL MATERIAL**

**Estimating the potential value of MSM-focused evidence-based implementation interventions in three Ending the HIV Epidemic jurisdictions in the U.S.: a model-based analysis**

Benjamin Enns^1^, Yi Sui^1^, Brenda C. Guerra-Alejos^1^, Lia Humphrey^1^, Micah Piske^1^, Xiao Zang^2^, Susanne Doblecki-Lewis^3^, Daniel J. Feaster^4^, Victoria A. Frye^5^, Elvin H. Geng^6^, Albert Y. Liu^7^, Brandon D.L. Marshall^8^, Scott D. Rhodes^9^, Patrick S. Sullivan^10^, Bohdan Nosyk^1,11§^ **On behalf of the localized economic modelling study group.**

1. Centre for Advancing Health Outcomes; Vancouver, British Columbia, Canada.

2. School of Public Health, University of Minnesota; Minneapolis, Minnesota, USA.

3. Division of Infectious Diseases, University of Miami Miller School of Medicine, Miami, Florida, USA

4. Department of Public Health Sciences, University of Miami Miller School of Medicine, Miami, Florida, USA

5. School of Social Work, Columbia University, New York, New York, USA

6. Center for Dissemination and Implementation, Institute of Public Health, Division of Infectious Diseases, Department of Medicine, School of Medicine, Washington University in St. Louis, St. Louis, Missouri, USA.

7. Bridge HIV, San Francisco Department of Public Health, San Francisco, California, USA

8. Department of Epidemiology, School of Public Health, Brown University, Providence, Rhode Island, USA

9. Department of Social Sciences and Health Policy, Wake Forest University School of Medicine, Winston-Salem, North Carolina, USA

10. School of Public Health, Emory University, Atlanta, Georgia, USA

11. Faculty of Health Sciences, Simon Fraser University; Burnaby, British Columbia, Canada.

**Table of contents:**

1. **Implementation interventions for scale-up of opt-out HIV testing delivered in primary care**
   1. Academic detailing for HIV testing
   2. CyBER/testing
   3. All About Me
2. **Implementation interventions for scale-up of pre-exposure prophylaxis (PrEP)**
   1. Project SLIP
   2. PrEPmate
   3. PrEP patient navigation

**Background**

**Model structure and dynamics**

To model the population-level impact of implementation interventions, we used a dynamic, compartmental HIV transmission model (**Figure S1**). Individuals aged 15-64 in each jurisdiction could transition between model health states in monthly cycles. The model explicitly captured heterogeneity in the risk of HIV transmission, maturation, mortality, and the disparities in service access (HIV testing, ART, syringe services programs, medication for opioid use disorder, and pre-exposure prophylaxis among men-who-have-sex-with-men [MSM]). The calibration and validation manuscript includes a more detailed description of the model structure and calibration,(1) and the evidence synthesis includes information on all model parameters for each of the 42 strata (men who have sex with men (MSM): 6 groups; people who inject drugs (PWID): 12 groups; MSM who inject drugs (MWID): 12 groups; and heterosexuals (HET): 12 groups) included in the model, and health resource use cost estimates included costs of antiretroviral treatment (ART) when applicable(2, 3). Sexual risk (high/low) was defined by exposure or behaviour that increases the probability of acquiring HIV. We used the variables found in the National HIV Behavioral Surveillance System of MSM reporting condom-less sex with casual partners for MSM.(1)

**Interventions**

Opt-out HIV testing in primary care and PrEP among indicated MSM were the existing focal interventions to which we applied implementation interventions across different jurisdictions. These are detailed in Krebs et al. (2020),(4) chosen for their promising scalability, higher potential for population-level impact, and consequently more potential barriers to successful implementation (e.g., integration into routine primary care for HIV testing, or medication adherence for PrEP). We did not explore implementation interventions focused on ART treatment engagement and re-engagement, as these were shown to have lower incremental value than interventions focused on testing and PrEP.(4)

**Cost-effectiveness analysis**

For each intervention, implementation costs accrued at the targeted scale of delivery during the first 18 months, sustainment costs accrued during the period following implementation and up to 10 years, and delivery costs accrued throughout the entire 10-year delivery period. Individual cost components were included when applicable as not all interventions had costs for each component. The delivery costs included costs specific to providing each intervention to the targeted individuals (e.g., personnel time required). Projected costs of interventions plus implementation interventions included the costs of delivering interventions at higher scale (e.g., more HIV tests, PrEP medication costs, etc.), as well as costs specific to implementation interventions. We derived costs for salaries from the Bureau of Labor Statistics (BLS),(5) included fringe benefits of 30.5% based on national BLS estimates (**Table S5**),(6) and multiplied by the estimated full-time equivalent (FTE) required for each component of an intervention. We estimated clinic staffing costs per physician FTE based on estimates in Peikes et al. (2014) (**Table S6**).(7) All costs are reported in 2022 USD, and any costs that had been reported in prior years were adjusted to 2022 USD using the U.S. consumer price index (CPI).(8) Conforming to best practice guidelines on cost-effectiveness analyses,(9) Supplemental **Tables S7** and **S8** report the Impact Inventory and the Consolidated Health Economic Evaluation Reporting Standards (CHEERS) checklist.

1. **Implementation interventions for scale-up of opt-out HIV testing delivered in primary care**

*Calculation of costs for HIV testing interventions*

We estimated the costs attributable to implementation interventions for HIV testing (**Table S3**) and PrEP (**Table S4**) using a framework informed by studies identified in a recent review,(10) as well as feedback on our estimates from the authors of the source studies. We estimated full-time equivalent labour requirements for each intervention, combined with salary estimates from the Bureau of Labor Statistics (**Table S5**),(11) clinic staffing costs based on estimates in Peikes et al. (2014) (**Table S6**);(7) as well as other non-labour costs specific to each intervention. Costs included implementation (accrued in scale-up period), delivery (accrued throughout intervention period to each individual reached), and sustainment (accrued during the period following implementation), though not all costs were applicable for all interventions. We also consulted with the authors of the source studies to verify costing and population-level scale assumptions, or provide additional information (**Table S10**).

We applied implementation costs (clinic-level fixed costs during scale-up) to the number of individuals tested, prorated over the 18-month scale-up period. Costs were accumulated per additional individual tested, and we prorated testing costs (with the exception of intervention delivery time spent with individuals prior to testing) by the estimated number of tests per clinic (Academic detailing for HIV testing) and tests per Health educator (CyBER/testing) and tests per recruiter (All About Me) (**Table S9**). Testing volumes used in this calculation do not reflect the model projected tests, but rather the potential individuals tested per month in the clinics, or by the staff described in each intervention.

- 1. **HIV testing implementation interventions – Academic detailing for HIV testing**
     1. *Description*

We derived estimates for this implementation intervention from Lubelchek et al. (2013).(12) Changes in HIV testing rates were measured in each clinic following the intervention. The intervention was sponsored by the Chicago Developmental Center for AIDS Research (D-CFAR) and the Chicago Department of Public Health.

- - 1. *Effectiveness and scale of delivery*

We derived population-level effectiveness for improving adoption based on the average effectiveness estimated in the trial across settings (average 30.0% increase in proportion of individuals tested).(12) We multiplied effectiveness by the percentage of providers who attended presentation, and multiplied by the percentage who responded to the survey overall (26.0% attended * 83.0% responded) (**Table S2**). Finally, we scaled effectiveness by structural constraints (MSM reporting health insurance coverage), which limited the potential scale of testing interventions. Together, we estimated an overall 4.6% increase in adoption among primary care providers for Atlanta and Miami, and 5.7% in Los Angeles (**Table 2**).

- - 1. *Costs*

We derived implementation costs according to the components noted in the intervention description, combined with person-time estimates. We estimated costs for survey development, administration, analysis, and costs to develop and deliver training presentations to providers (**Table S3**). Costs entered the model as a cost per additional individual tested, so clinic-level costs were prorated over the average monthly number of individuals tested in each clinic, as reported in Lubelchek et al. (2013) (**Table S9**).(12) We assumed no additional delivery or sustainment costs once the intervention had been delivered.

- 1. **HIV testing implementation interventions – CyBER/testing**
     1. *Description*

This implementation intervention involved the promotion of HIV testing on commonly used social media platforms among MSM and transgender persons, as reported in Rhodes et al. (2016).(13) The platforms include: Adam4Adam, BlackGayChat, Craigslist, and Gay.com. Participants were given an opportunity to complete an assessment, following the intervention to assess changes in testing behaviour (primary study outcome was past 12-month odds of HIV testing at post-test compared to baseline). This study was supported by a National Institute of Mental Health grant.

- - 1. *Scale of delivery*

We derived population-level effectiveness for improving reach based on effectiveness in the trial setting: 34.6% of intervention participants reported past 12-month HIV testing compared with 38.5% of comparison participants. At post-test, 63.7% of intervention participants reported past 12-month HIV testing compared with 42.0% of comparison participants (74.0% increase attributable to intervention) (**Table S2**).(13) We scaled this effect for reaching the full target population by the proportion of individuals who reported using dating websites or apps to meet other men in the past 12 months (‘awareness’ = 66.5%),(14) combined with the proportion of MSM who reported that they wanted to receive sexual health information via apps (‘acceptability’ = 63.8%).(15) Thus, we estimated an overall 18.6% increase in reach of testing among MSM in Atlanta and Miami, and 22.8% in Los Angeles (**Table 2**).

- - 1. *Costs*

Given that this intervention was delivered on existing platforms we assumed no overhead implementation costs, such as launching or hosting websites/apps, and assumed that person-time costs of health educators were the only cost of the intervention. We excluded costs of participant feedback, as this was specifically done for the trial. We assumed no additional delivery or sustainment costs. Costs entered the model as a cost per additional individual tested, so we prorated costs by the estimated number of monthly individuals tested per health educator (**Table S9**).

- 1. **HIV testing implementation interventions – All About Me**
     1. *Description*

This implementation intervention by Frye et al. (2020)(16) was used to derive estimates of optimal HIV testing options (e.g. self-testing, clinic-based tests, HIV counseling). For the intervention effectiveness input in the model, we estimated the increase in testing based on the overall increase across both intervention and control arms (which could be attributed to the information provided and increased awareness of HIV testing). We chose to model the overall effects of providing information on testing, as both groups had near-equal increases over baseline. We applied the estimated effectiveness of this intervention to the reach component of HIV testing delivered in primary care clinics, applied to black MSM. This study was grant-funded by the Eunice Kennedy Shriver National Institute of Child Health and Human Development

- - 1. *Scale of delivery*

We estimated the population-level scaling factor for the extent to which the effectiveness of this intervention could be delivered at a population level. We scaled the effect of improvement by the estimated proportion of MSM aged 16-29 (note that black MSM are captured explicitly in the model via race and HIV risk stratification), which we estimated to be 39.0% based on 2017 NHBS estimates of 10,104 MSM.(17) We multiplied this by the proportion of participants who were recommended to clinic-based testing during the trial (as the focus of this analysis was specifically on improvements to testing delivered in primary care clinics). We assumed that structural barriers limiting potential intervention uptake would be the proportion of MSM with insurance coverage in the US (estimated by census region).(18) Thus, we estimated an overall 8.2% increase in reach of testing among MSM in Atlanta and Miami, and 10.0% in Los Angeles (**Table 2**).

- - 1. *Costs*

Implementation costs are incurred as one-time start-up expenditures for the intervention, delivery costs are incurred per individual tested, and sustainment costs are recurring costs for the duration of the intervention (**Table S3**). These include intervention administration, developing survey and informational materials, and building and maintaining web-based platforms. We excluded costs that were specific to the trial, and wouldn’t be incurred when scaling up to a larger population (e.g., incentives for participation). We scaled resource requirements by the proportion of individuals directed to clinic-based testing; however, costs and resource requirements were estimated at a jurisdiction-level as this intervention can be delivered at a larger geographic area. Intervention costs entered the model as a cost per additional individual tested, so we prorated costs (with the exception of recruiter time spent with individuals) by the estimated number of individuals who could be seen by one recruiter (**Table S9**).

1. **Implementation interventions to increase scale-up of PrEP**

*Calculation of costs for PrEP interventions*

For costing of PrEP implementation interventions, we applied implementation costs (clinic-level fixed costs during scale-up) to the incremental number of individuals receiving PrEP, prorated over the 18-month scale-up period. Implementation cost estimates were derived at the provider/clinic-level, and prorated over the average number of patients-per-provider (22 [Atlanta], 17 [Los Angeles], 30 [Miami]),(19) and providers-per-clinic (median estimate of 3 providers per clinic) estimated in Peikes et al. (2014),(7) for a total of 67, 51, and 89 PrEP patients per clinic estimated for Atlanta, Los Angeles, and Miami, respectively. When applicable, we applied sustainment costs over the duration of the intervention period spread over all additional PrEP patients in the intervention scenario vs. our comparator scenario, while delivery costs of implementation interventions were attributed only to new PrEP patients each month, as these were variable costs related to recruitment of new PrEP patients and would not apply to those already on PrEP (i.e., individuals receiving PrEP at time (t) – individuals receiving PrEP at time (t – 1), within the intervention scenario). We derived costs based on estimated person-hour requirements to complete each component, multiplied by hourly salary estimates from the U.S. Bureau of Labor Statistics (BLS) for specific job titles, as well as any additional materials costs or other overhead (note that PrEP medication and wraparound services such as HIV testing are already captured in the model, and not included as intervention-specific costs – however, differences in these costs resulting from more individuals on PrEP are still captured between scenarios).

- 1. **Implementation interventions to improve PrEP – Project SLIP**
     1. *Description*

We derived estimates from a study on the effectiveness of eligibility screening questionnaires and linkage in primary care settings detailed in Storholm et al. (2021).(20) We applied the estimated effectiveness of this intervention to the adoption component of existing PrEP access. This study was funded by the National Institutes of Health.

- - 1. *Scale of delivery*

We derived population-level effectiveness based on effectiveness in the trial setting [+16.9% increase in individuals on PrEP: 22.0% (increase in PrEP referrals) * 77.0% (PrEP prescription fills among referrals)]. We multiplied by an intervention scaling factor for reaching the full target population [10.8% (number of referrals compared to the total number of eligible patients)]. To account for structural barriers, we scaled intervention effectiveness by the proportion of MSM who reported current insurance coverage in 2017 for ACA (Los Angeles) and non-ACA states (Atlanta, Miami); 87.9% and 71.6%.(18) This resulted in an overall increase of 1.3% (Atlanta, Miami) and 1.6% (Los Angeles) for PrEP adoption among MSM (**Table 2**).

- - 1. *Costs*

We derived implementation costs based on estimated time required for developing training presentations; conducting initial training sessions (for all clinic staff) as well as with individual providers; developing survey materials, as well as eliciting survey feedback from staff (**Table S4**). Delivery costs for the intervention included time costs to distribute and collect surveys, which were prorated based on the number of surveys delivered per individual who initiated PrEP (17 prescribed PrEP vs. 1225 total referrals from Storholm et al. [2021]).(20)

- 1. **Implementation interventions to improve PrEP – PrEPmate**
     1. *Description*

We derived effect estimates on increased PrEP effectiveness from a study on a text messaging intervention on study retention and PrEP adherence for young MSM (Liu et al. [2019]).(21) For the intervention effectiveness input in the model, we estimated the increase in PrEP adherence based on the improved adherence for intervention versus control groups. We applied the estimated effectiveness of this intervention to the reach component of PrEP scale-up among MSM (as a proxy for retention). This study was funded by the National Institutes of Mental Health, with a private research company providing study drug and testing for adherence measures.

- - 1. *Scale of delivery*

We derived population-level effectiveness based on estimated trial effectiveness; +28.2% increase in effectiveness (adherence entered the model as percentage of individuals maintaining protective adherence, so we used a risk-ratio approximation to estimate the corresponding percentage increase in adherence based on the adjusted odds-ratio estimate of 2.06 in the trial study, and baseline probability of 57.0% in control group) for improved retention and adherence to PrEP among those who are already receiving PrEP, multiplied by a scaling factor for reaching the full target population (91.0%; proportion of MSM enrolled among screened in trial). We did not scale effectiveness improvements (improved adherence) by insurance status, however, as this applied only to individuals who were actively receiving PrEP and would not be affected by insurance status. This resulted in an increase of 25.7% in PrEP effectiveness across all jurisdictions (**Table 2**).

- - 1. *Costs*

We derived implementation costs based on the estimated costs for setting up a text message reminder system at a clinic (we assumed that clinics would not have existing services, however, these reminders could be integrated within existing systems which would reduce the cost) (**Table S4**). We also estimated costs for developing training presentations, as well as delivering these presentations at clinics. We assumed no continuous delivery costs once systems were set up, and we included ongoing subscription and maintenance costs as intervention sustainment costs.

- 1. **Implementation interventions to improve PrEP – PrEP patient navigation**
     1. *Description*

Our source study was an intervention for PrEP linkage by an individualized approach of community-based case management by Doblecki-Lewis et al. (2019),(22) evaluating PrEP initiation and time to initiation. For the intervention effectiveness input in the model, we estimated the increase in PrEP reach based on differences in PrEP initiation between treatment and control groups after 12 weeks. We applied the estimated effectiveness of this intervention to the reach component of PrEP scale-up among indicated MSM. This study was funded by a Miami Center for AIDS Research Administrative Supplement.

- - 1. *Scale of delivery*

We derived population-level effectiveness based on estimated trial effectiveness (+21.0% [difference in PrEP initiation between treatment and control groups at 12 weeks]),(22) multiplied by a scaling factor for reaching the full target population (number of participants vs. the total number of eligible individuals; 55.0%). We scaled intervention effectiveness by the proportion of MSM who reported some insurance coverage in ACA (Los Angeles) and non-ACA states (Atlanta, Miami); 87.9% and 71.6%. Together, this resulted in a population-level increase of 8.3% in PrEP reach (Atlanta, Miami) and 10.2% in Los Angeles (**Table 2**).

- - 1. *Costs*

We derived implementation costs based on the estimated costs of training patient navigators at a clinic (including one trainer/program director), administration, and patient navigator visit, as well as overhead for office space and cell phone access for patient navigators (**Table S4**). We assumed a caseload of 75 individuals per patient-navigator, and five patient navigators per clinic. Costs were derived per individual initiating PrEP.

**References**

1. Zang X, Krebs E, Min JE, Pandya A, Marshall BDL, Schackman BR, et al. Development and Calibration of a Dynamic HIV Transmission Model for 6 US Cities. Med Decis Making. 2020;40(1):3-16.

2. Enns B, Krebs E, Mathews WC, Moore RD, Gebo KA, Nosyk B. Heterogeneity in the costs of medical care among people living with HIV/AIDS in the United States. Aids. 2019;33(9):1491-500.

3. Krebs E, Panagiotoglou D, Wang L, Enns B, Zang X, Del Rio C, et al. Dynamic Transmission Model for 6 U.S. Cities: An Evidence Synthesis. PLoS ONE. 2019;14(5):e0217559.

4. Krebs E, Zang X, Enns B, Min JE, Behrends CN, Del Rio C, et al. The impact of localized implementation: determining the cost-effectiveness of HIV prevention and care interventions across six United States cities. AIDS. 2020;34(3):447-58.

5. Bureau of Labor Statistics. May 2017 National Occupational Employment and Wage Estimates: United States 2018 [Available from: <https://www.bls.gov/oes/current/oes_nat.htm#29-0000> [Accessed February 14, 2019].

6. Bureau of Labor Statistics. Employer Costs for Employee Compensation for the Regions - March 2018 2018 [Available from: <https://www.bls.gov/regions/southwest/news-release/employercostsforemployeecompensation_regions.htm> [Accessed September 12, 2019].

7. Peikes DN, Reid RJ, Day TJ, Cornwell DD, Dale SB, Baron RJ, et al. Staffing patterns of primary care practices in the comprehensive primary care initiative. Ann Fam Med. 2014;12(2):142-9.

8. Bureau of Labor Statistics. Consumer Price Index 2019 [Available from: <https://www.bls.gov/cpi/> [Accessed February 14, 2019].

9. Sanders GD, Neumann PJ, Basu A, Brock DW, Feeny D, Krahn M, et al. Recommendations for conduct, methodological practices, and reporting of cost-effectiveness analyses: Second Panel on Cost-Effectiveness in Health and Medicine. JAMA. 2016;316(10):1093.

10. Michaud TL, Pereira E, Porter G, Golden C, Hill J, Kim J, et al. Scoping review of costs of implementation strategies in community, public health and healthcare settings. BMJ Open. 2022;12(6):e060785.

11. U.S. Bureau of Labor Statistics. Occupational Employment and Wage Statistics 2021 [Available from: <https://www.bls.gov/oes/home.htm>.

12. Lubelchek RJ, Hotton AL, Taussig D, Amarathithada D, Gonzalez M. Scaling up routine HIV testing at specialty clinics: assessing the effectiveness of an academic detailing approach. J Acquir Immune Defic Syndr. 2013;64 Suppl 1(0 1):S14-9.

13. Rhodes SD, McCoy TP, Tanner AE, Stowers J, Bachmann LH, Nguyen AL, et al. Using Social Media to Increase HIV Testing Among Gay and Bisexual Men, Other Men Who Have Sex With Men, and Transgender Persons: Outcomes From a Randomized Community Trial. Clin Infect Dis. 2016;62(11):1450-3.

14. Hecht J, Zlotorzynska M, Sanchez TH, Wohlfeiler D. Gay Dating App Users Support and Utilize Sexual Health Features on Apps. AIDS Behav. 2022;26(6):2081-90.

15. Sun CJ, Stowers J, Miller C, Bachmann LH, Rhodes SD. Acceptability and feasibility of using established geosocial and sexual networking mobile applications to promote HIV and STD testing among men who have sex with men. AIDS Behav. 2015;19(3):543-52.

16. Frye V, Nandi V, Hirshfield S, Chiasson MA, Wilton L, Usher D, et al. Brief Report: Randomized Controlled Trial of an Intervention to Match Young Black Men and Transwomen Who Have Sex With Men or Transwomen to HIV Testing Options in New York City (All About Me). J Acquir Immune Defic Syndr. 2020;83(1):31-6.

17. Cha S, Xia M, Finlayson T, lainn Sionean C, Teplinskaya A, Morris E, et al. HIV Infection risk, prevention, and testing behaviors among men who have sex with men National HIV behavioral surveillance 23 US cities, 2017. 2019.

18. Baugher AR, Finlayson T, Lewis R, Sionean C, Whiteman A, Wejnert C, et al. Health Care Coverage and Preexposure Prophylaxis (PrEP) Use Among Men Who Have Sex With Men Living in 22 US Cities With vs Without Medicaid Expansion, 2017. Am J Public Health. 2021;111(4):743-51.

19. Zhu W, Huang YA, Kourtis AP, Hoover KW. Trends in the Number and Characteristics of HIV Pre-Exposure Prophylaxis Providers in the United States, 2014-2019. J Acquir Immune Defic Syndr. 2021;88(3):282-9.

20. Storholm ED, Siconolfi D, Huang W, Towner W, Grant DL, Martos A, et al. Project SLIP: Implementation of a PrEP Screening and Linkage Intervention in Primary Care. AIDS Behav. 2021;25(8):2348-57.

21. Liu AY, Vittinghoff E, von Felten P, Rivet Amico K, Anderson PL, Lester R, et al. Randomized Controlled Trial of a Mobile Health Intervention to Promote Retention and Adherence to Preexposure Prophylaxis Among Young People at Risk for Human Immunodeficiency Virus: The EPIC Study. Clin Infect Dis. 2019;68(12):2010-7.

22. Doblecki-Lewis S, Butts S, Botero V, Klose K, Cardenas G, Feaster D. A Randomized Study of Passive versus Active PrEP Patient Navigation for a Heterogeneous Population at Risk for HIV in South Florida. J Int Assoc Provid AIDS Care. 2019;18:2325958219848848.

**Tables and figures

Table S1. Definitions and assumptions of the RE-AIM framework used for modelling implementation.**

**Table S2. Population and intervention-specific scaling limits for population-level effectiveness of implementation interventions.**

| **Implementation Intervention** | **Target RE-AIM component** | **Intervention-based scale limit** | **Structural barrier scale limit** | **Total population-level scale limit** |
| --- | --- | --- | --- | --- |
| *Implementation interventions for scale-up of opt-out HIV testing delivered in primary care* | | |  |  |
| Academic detailing for HIV testing | Adoption | Scale limit: 21.6%; Percentage of providers responding to survey (83.0%) * Percentage of respondents attending workshop (26.0%) | Scale limit: 71.6% (ATL & MIA); 87.9% (LA) (Percentage of MSM reporting insurance coverage by region) | Combined scale limit: 15.5% (ATL & MIA); 19.0% (LA) |
| CyBER/testing | Reach | Scale limit: 42.4%; Awareness: 6,737/10,129 (66.5%) of AMIS-2018 survey respondents reported using dating websites or apps * Acceptability: 63.8% of study participants reported wanting to receive sexual health information via apps | Scale limit: 71.6% (ATL & MIA); 87.9% (LA) (Percentage of MSM reporting insurance coverage by region) | Combined scale limit: 30.4% (ATL & MIA); 37.3% (LA) |
| All About Me | Reach | Scale limit: 16.8%; Proportion of Black MSM aged 16-29 from NHBS survey 2017 * 43.2% of participants recommended to clinic-based testing (Frye et al. 2020). | Scale limit: 71.6% (ATL & MIA); 87.9% (LA) (Percentage of MSM reporting insurance coverage by region) | Combined scale limit: 12.1% (ATL & MIA); 14.8% (LA) |
| *Implementation interventions to increase scale-up of PrEP* | | |  |  |
| Project SLIP | Adoption | Scale limit: 10.8% (132/1,225 referred among all eligible) | Scale limit: 71.6% (ATL & MIA); 87.9% (LA) (Percentage of MSM reporting insurance coverage by region) | Combined scale limit: 7.7% (ATL & MIA); 9.5% (LA) |
| PrEPmate | Effectiveness | Scale limit: 91.0% (trial participation, proportion enrolled among screened [121/134]) | Scale limit: Insurance-based scaling factors were not applied to PrEP adherence (effectiveness) estimates, as this was only applied to individuals already receiving PrEP. | Combined scale limit (Effectiveness): 91.0% (All cities) |
| PrEP patient navigation | Reach | Scale limit: 55.0% [trial participation (61/110 participants assessed were recruited)] | Scale limit: 71.6% (ATL & MIA); 87.9% (LA) (Percentage of MSM reporting insurance coverage by region) | Combined scale limit: 39.4% (ATL & MIA); 48.3% (LA) |

RE-AIM – reach, effectiveness, adoption, implementation, maintenance; ATL – Atlanta; LA – Los Angeles; MIA – Miami; MSM - Men who have sex with men; CyBER – Cyber-Based Education and Referral; AMIS - American Men’s Internet Survey; NHBS – National HIV Behavioral Surveillance; SLIP - Screening and Linkage Intervention in Primary Care.

**Table S3. HIV testing implementation intervention cost estimates.**

|  |  |  | **Additional monthly clinic costs** | | |  | **Additional monthly costs per individual tested** | | |
| --- | --- | --- | --- | --- | --- | --- | --- | --- | --- |
| **Cost component of testing implementation interventions** | **Cost type**† | **Time (hr)** | **$/hr (2022 USD)** | | |  | **$ (2022 USD)** | | |
|  |  |  | **Atlanta** | **Los Angeles** | **Miami** |  | **Atlanta** | **Los Angeles** | **Miami** |
| ***Academic detailing for HIV testing*** |  |  |  |  |  |  |  |  |  |
| Intervention administration (clerical, HR, etc.) | I; S | 41 | $25.19 | $31.16 | $24.96 |  | $3.65 | $4.51 | $3.61 |
| Develop training presentation (program director) | I | 6 | $67.47 | $74.57 | $65.81 |  | $1.44 | $1.59 | $1.41 |
| Initial training session (1 program director + all clinic staff) | I | 1 | $1,292.00 | $1,454.00 | $1,185.00 |  | $4.60 | $5.18 | $4.22 |
| Provider training (1 program director + physician) | I | 1 | $248.00 | $231.00 | $219.00 |  | $0.88 | $0.82 | $0.78 |
| Develop survey materials (1 coordinator) | I | 6 | $19.93 | $41.00 | $40.31 |  | $0.43 | $0.88 | $0.86 |
| Feedback on survey from staff | I | 0.25 | $1,225.00 | $1,379.50 | $1,118.71 |  | $1.09 | $1.23 | $1.00 |
|  |  |  |  |  |  |  |  |  |  |
| Subtotal | I |  | -- | -- | -- |  | $8.45 | $9.70 | $8.27 |
| Subtotal | D |  | -- | -- | -- |  | $0.00 | $0.00 | $0.00 |
| Subtotal | S |  | -- | -- | -- |  | $3.65 | $4.51 | $3.61 |
| ***CyBER/testing*** |  |  |  |  |  |  |  |  |  |
| Community organizations | I; S | -- | -- | -- | -- |  | $0.62 | $0.62 | $0.62 |
| Intervention administration | D | -- | -- | -- | -- |  | $1.03 | $1.03 | $1.03 |
| Executive director/supervision | D | -- | -- | -- | -- |  | $1.55 | $1.55 | $1.55 |
| Health educator | D | -- | -- | -- | -- |  | $3.10 | $3.10 | $3.10 |
| Local travel | D | -- | -- | -- | -- |  | $0.12 | $0.12 | $0.12 |
| Space | D | -- | -- | -- | -- |  | $0.45 | $0.45 | $0.45 |
| Telephone and internet | D | -- | -- | -- | -- |  | $0.15 | $0.15 | $0.15 |
|  |  |  |  |  |  |  |  |  |  |
| Subtotal | I | -- | -- | -- | -- |  | $0.62 | $0.62 | $0.62 |
| Subtotal | D | -- | -- | -- | -- |  | $6.40 | $6.40 | $6.40 |
| Subtotal | S | -- | -- | -- | -- |  | $0.62 | $0.62 | $0.62 |
| ***All About Me*** |  |  |  |  |  |  |  |  |  |
| Intervention administration (clerical, HR, etc.) | I; S | 163 | $25.19 | $31.16 | $24.96 |  | $0.34 | $0.42 | $0.34 |
| Develop training presentation (program director) | I | 6 | $67.47 | $74.57 | $65.81 |  | $0.03 | $0.04 | $0.03 |
| Recruitment (via online websites) + information | D | 0.25 | $22.33 | $25.25 | $24.83 |  | $5.58 | $6.31 | $6.21 |
| Online survey hosting | D | -- | -- | -- | -- |  | $0.16 | $0.16 | $0.16 |
| Telephone and internet | D | -- | -- | -- | -- |  | $0.30 | $0.30 | $0.30 |
|  |  |  |  |  |  |  |  |  |  |
| Subtotal | I |  | -- | -- | -- |  | $0.37 | $0.46 | $0.37 |
| Subtotal | D |  | -- | -- | -- |  | $6.04 | $6.77 | $6.67 |
| Subtotal | S |  | -- | -- | -- |  | $0.34 | $0.42 | $0.34 |

USD – United States Dollar; HIV - human immunodeficiency virus; HR – human resources; CyBER: Cyber-Based Education and Referral.
† I – implementation; S – sustainment; D – delivery.

**Table S4. PrEP implementation intervention cost estimates.**

|  |  | **Clinic Costs Per Physician** | | | |  | **Monthly costs per individual** | | |
| --- | --- | --- | --- | --- | --- | --- | --- | --- | --- |
|  |  | **Time (hr)** | **$/hr (2022 USD)** | | |  | **$ (2022 USD)** | | |
| **Cost component of PrEP implementation interventions** | **Cost type†** |  | **Atlanta** | **Los Angeles** | **Miami** |  | **Atlanta** | **Los Angeles** | **Miami** |
| ***Project SLIP*** |  |  |  |  |  |  |  |  |  |
| Intervention administration (clerical, HR, etc.) | I; S | 41 | $23.79 | $29.43 | $23.57 |  | $43.21 | $70.56 | $32.22 |
| Develop training presentation (program director) | I | 6 | $64.00 | $70.00 | $62.00 |  | $0.95 | $1.39 | $0.70 |
| Initial training session (1 program director + all clinic staff) | I | 1 | $1,220.00 | $1,373.00 | $1,119.00 |  | $3.03 | $4.50 | $2.09 |
| Provider training | I | 1 | $170.46 | $147.84 | $144.79 |  | $0.42 | $0.48 | $0.27 |
| Develop survey materials (1 coordinator) | I | 6 | $18.82 | $38.72 | $38.07 |  | $0.28 | $0.76 | $0.43 |
| Feedback on survey from staff | I | 0.25 | $1,156.74 | $1,302.64 | $1,056.38 |  | $0.72 | $1.07 | $0.49 |
| Distribute/collect survey (1 RN) | D | 0.25 | $48.74 | $75.44 | $48.32 |  | $878.57 | $1,359.84 | $871.04 |
|  |  |  |  |  |  |  |  |  |  |
| Subtotal | I |  | -- | -- | -- |  | $5.40 | $8.20 | $3.98 |
| Subtotal | D |  | -- | -- | -- |  | $878.57 | $1,359.84 | $871.04 |
| Subtotal | S |  | -- | -- | -- |  | $43.21 | $70.56 | $32.22 |
| ***PrEPmate*** |  |  |  |  |  |  |  |  |  |
| Intervention administration (clerical, HR, etc.) | I; S | 8 | $23.79 | $29.43 | $23.57 |  | $8.64 | $14.11 | $6.44 |
| Text reminder setup (one-time) | I |  |  |  |  |  | $0.83 | $1.09 | $0.62 |
| Develop training presentation (program director) | I | 6 | $64.00 | $70.00 | $62.00 |  | $0.95 | $1.39 | $0.70 |
| Training (1 program director + all clinic staff) | I | 1 | $1,220.00 | $1,373.00 | $1,119.00 |  | $3.03 | $4.50 | $2.09 |
| Subscription and maintenance (monthly) | S |  |  |  |  |  | $6.25 | $6.25 | $6.25 |
|  |  |  |  |  |  |  |  |  |  |
| Subtotal | I |  | -- | -- | -- |  | $4.81 | $6.98 | $3.41 |
| Subtotal | D |  | -- | -- | -- |  | $0.00 | $0.00 | $0.00 |
| Subtotal | S |  | -- | -- | -- |  | $14.89 | $20.36 | $12.69 |
| ***PrEP patient navigation*** |  |  |  |  |  |  |  |  |  |
| Intervention administration (clerical, HR, etc.) | I; S | 41 | $23.79 | $29.43 | $23.57 |  | $43.21 | $70.56 | $32.22 |
| Patient navigator training (1 PN + 1 trainer/program director) | I | 4 | $84.80 | $94.26 | $85.59 |  | $0.25 | $0.28 | $0.25 |
| Patient navigator visit [1 PN (Median (Q1-Q3): 2 (1-5))] | D | 2 | $21.09 | $23.84 | $23.45 |  | $35.15 | $39.74 | $39.08 |
| Space | D | -- | -- | -- | -- |  | $1.60 | $1.60 | $1.60 |
| Cellular phone for patient navigators | D | -- | -- | -- | -- |  | $1.33 | $1.33 | $1.33 |
|  |  |  |  |  |  |  |  |  |  |
| Subtotal | I |  | -- | -- | -- |  | $43.46 | $70.84 | $32.48 |
| Subtotal | D |  | -- | -- | -- |  | $38.08 | $42.67 | $42.02 |
| Subtotal | S |  | -- | -- | -- |  | $43.21 | $70.56 | $32.22 |

PrEP – pre-exposure prophylaxis; USD – United States Dollar; SLIP - Screening and Linkage Intervention in Primary Care; HR – human resources; RN – registered nurse; PN – patient navigator.
† I – implementation; S – sustainment; D – delivery.

**Table S5. Staff salary estimates from Bureau of Labor Statistics.**

| **Staff Type** | **BLS Salary ($/hour)** | | | **BLS Title** |
| --- | --- | --- | --- | --- |
|  | **Atlanta** | **Los Angeles** | **Miami** |  |
| Program director (PD) | $67.47 | $74.57 | $65.81 | Medical and Health Services Managers |
| Coordinator | $19.93 | $41.00 | $40.31 | Healthcare Support Occupations |
| Physician | $180.52 | $156.57 | $153.33 | Physicians, All Other |
| Nurse | $51.62 | $79.89 | $51.18 | Registered Nurses |
| IT staff | $107.74 | $109.01 | $87.88 | Computer and Information Systems Managers |
| Patient navigator | $22.33 | $25.25 | $24.83 | Healthcare Support Workers, All Other |
| Administrative support (clerical, HR, etc.) | $25.19 | $31.16 | $24.96 | Medical Secretaries and Administrative Assistants |
| All clinic staff (per physician) | $1,225.00 | $1,379.50 | $1,118.71 |  |

BLS – Bureau of Labor Statistics; IT – information technology; HR – human resources.

**Table S6. Clinic staffing estimates.**

|  | **Staff per physician (headcount)** | | | | |  | **BLS salary ($/hour)** | | | **Staff cost per physician ($/hour)** | | | **Staff cost per clinic ($/hour)** | | |
| --- | --- | --- | --- | --- | --- | --- | --- | --- | --- | --- | --- | --- | --- | --- | --- |
| **Staff type** | **≤2 FTE** | **>2-4 FTE** | **>4-7 FTE** | **>7 FTE** | **Average Physician** | **Average Clinic** | **ATL** | **LA** | **MIA** | **ATL** | **LA** | **MIA** | **ATL** | **LA** | **MIA** |
| *Median number of physicians* | 1.00 | 3.00 | 5.00 | -- | 3.00 | 3.00 | $130.62 | $113.29 | $110.95 | $131 | $113 | $111 | $392 | $340 | $333 |
| **Clinic staff per physician FTE** |  |  |  |  |  |  |  |  |  |  |  |  |  |  |  |
| *Administrative* | 2.42 | 1.76 | 1.70 | 1.98 | 2.05 | 6.15 | $18.13 | $22.69 | $17.79 | $37 | $47 | $36 | $111 | $140 | $109 |
| *Medical Assistants* | 1.76 | 1.31 | 1.23 | 1.11 | 1.45 | 4.35 | $17.76 | $17.98 | $14.07 | $36 | $37 | $29 | $109 | $111 | $87 |
| *NP/PA* | 0.97 | 0.49 | 0.38 | 0.20 | 0.65 | 1.95 | $49.22 | $63.72 | $49.11 | $101 | $131 | $101 | $303 | $392 | $302 |
| *LPN/LVN* | 1.38 | 0.78 | 0.66 | 0.53 | 0.95 | 2.85 | $23.25 | $29.47 | $22.72 | $48 | $60 | $47 | $143 | $181 | $140 |
| *RN* | 1.04 | 0.54 | 0.38 | 0.31 | 0.64 | 1.92 | $37.35 | $57.81 | $37.03 | $77 | $119 | $76 | $230 | $356 | $228 |
| *Care coordinator* | 0.77 | 0.46 | 0.24 | 0.23 | 0.47 | 1.41 | $36.08 | $47.76 | $30.34 | $74 | $98 | $62 | $222 | $294 | $187 |
| *Pharmacist* | 0.75 | 0.42 | 0.15 | 0.29 | 0.32 | 0.96 | $61.86 | $63.25 | $61.13 | $127 | $130 | $125 | $380 | $389 | $376 |
| *Social worker* | 0.75 | 0.22 | 0.13 | 0.12 | 0.20 | 0.60 | $26.78 | $38.34 | $29.63 | $55 | $79 | $61 | $165 | $236 | $182 |
| *Community service coordinator* | 0.86 | 0.26 | 0.17 | 0.20 | 0.48 | 1.44 | $22.59 | $22.79 | $19.15 | $46 | $47 | $39 | $139 | $140 | $118 |
| *Health educator* | 1.00 | 0.37 | 0.19 | 0.10 | 0.42 | 1.26 | $48.22 | $29.86 | $30.23 | $99 | $61 | $62 | $297 | $184 | $186 |
| *Nutritionist* | 0.58 | 0.38 | 0.08 | 0.07 | 0.27 | 0.81 | $27.43 | $37.99 | $29.55 | $56 | $78 | $61 | $169 | $234 | $182 |
| **Total** | 12.28 | 6.99 | 5.31 | 5.14 | **7.90** | **23.70** | -- | -- | -- | **$886** | **$998** | **$809** | **$2,659** | **$2,995** | **$2,428** |

BLS – Bureau of Labor Statistics; FTE – full-time equivalent; ATL – Atlanta; LA – Los Angeles; MIA – Miami; NP – nurse practitioner; PA – physician assistant; LPN – licensed practical nurse; LVN – licensed vocational nurse; RN – registered nurse.

**Table S7. Impact Inventory**

| **Sector** | **Type of Impact** | **Perspective** | | **Notes** |
| --- | --- | --- | --- | --- |
|  |  | **Third-Party Payer** | **Societal** |  |
| **Formal Health Care** | | | | |
| **Health** | **Health Outcomes (Effects)** | | | |
|  | **Longevity** |  | √ |  |
|  | **HRQoL** |  | √ |  |
|  | **Other Health Effects** |  | √ | Averted HIV acquisitions |
|  | **Medical Costs** | | | |
|  | **Third-Party Payers** |  | √ |  |
|  | **Patients out-of-pocket** |  | √ |  |
|  | **Future related medical costs** |  | √ |  |
|  | **Future unrelated medical costs** |  |  |  |
| **Informal Health Care** | | | | |
| **Health** | **Patient-time costs** | N/A |  |  |
|  | **Unpaid caregiver-time costs** | N/A |  |  |
|  | **Transportation costs** | N/A |  |  |
| **Non-Health Care Sectors** | | | | |
| **Productivity** | **Labour market earnings lost** | N/A |  |  |
|  | **Cost of unpaid lost productivity** | N/A |  |  |
|  | **Cost of uncompensated household production** | N/A |  |  |
| **Consumption** | **Future consumption unrelated to health** | N/A |  |  |
| **Social Services** | **Cost of social services related to intervention** | N/A |  |  |
| **Legal or criminal justice** | **Number of crimes related to intervention** | N/A |  |  |
|  | **Cost of crimes related to intervention** | N/A |  |  |
| **Education** | **Impact on educational achievement** | N/A |  |  |
| **Housing** | **Cost of intervention on home improvements** | N/A |  |  |
| **Environment** | **Production of toxic waste by intervention** | N/A |  |  |
| **Other** | **Other impacts** | N/A |  |  |

HRQoL: health-related quality of life; N/A: not available.

**Table S8. Consolidated Health Economic Evaluation Reporting Standards (CHEERS) Checklist.**

| **Item** | **Item** | **Recommendation** | **Section** |
| --- | --- | --- | --- |
| ***Title and Abstract*** |  |  |  |
| Title | 1 | Identify the study as an economic evaluation | Title |
| Abstract | 2 | Provide a structured summary of objectives, perspective, setting, methods, results, and conclusions | Abstract |
| ***Introduction*** |  |  |  |
| Background and objectives | 3 | Provide an explicit statement of the broader context for the study | Introduction |
| ***Methods*** |  |  |  |
| Target population and subgroups | 4 | Describe characteristics of the base case population and subgroups analysed, including why they were chosen | Methods: Model Description |
| Setting and location | 5 | State relevant aspects of the system in which decisions need to be made | Methods: Model Description |
| Study perspective | 6 | Describe the perspective of the study and relate this to the costs being evaluated | Methods: Cost-effectiveness Analysis |
| Comparators | 7 | Describe the interventions or strategies being compared and state why they were chosen | Methods: Interventions Assessed |
| Time horizon | 8 | State the time horizons over which costs and consequences are being evaluated | Methods: Cost-effectiveness Analysis |
| Discount rate | 9 | Report/explain the choice of discount rate used for costs and outcomes | Methods: Cost-effectiveness Analysis |
| Choice of health outcomes | 10 | Describe what outcomes were used as the measure of benefit in the evaluation and their relevance for the analysis | Methods: Cost-effectiveness Analysis |
| Measurement of effectiveness | 11 | Describe fully the methods used for identification of included studies and synthesis of clinical effectiveness data | Methods: Interventions Assessed; Evidence Verification; Supplemental Material |
| Measurement and valuation of preference based outcomes | 12 | If applicable, describe the population and methods used to elicit preferences for outcomes | N/A |
| Estimating resources and costs | 13 | Describe approaches and data sources used to estimate resource use associated with model health states | Supplemental Material |
| Currency, price date and conversion | 14 | Report the dates of the estimated resource quantities and unit costs | Methods: Cost-effectiveness Analysis; Supplemental Material |
| Choice of model | 15 | Describe and give reasons for the specific type of decision-analytical model used | Introduction |
| Assumptions | 16 | Describe all structural or other assumptions underpinning the decision-analytical model | Methods: Model Description; Supplemental Material |
| Analytical methods | 17 | Describe all analytical methods supporting the evaluation | Methods: Cost-effectiveness Analysis |
| ***Results*** |  |  |  |
| Study parameters | 18 | Report the values, ranges, references, and probability distributions for all parameters | Supplemental Material |
| Incremental costs and outcomes | 19 | For each intervention, report mean values for the main categories of estimated costs and outcomes of interest, as well as mean differences between comparator groups | Results; Supplemental Material |
| Characterising uncertainty | 20 | Describe the effects on the results of uncertainty for all input parameters and uncertainty related to the structure of the model and assumptions | Results; Discussion |
| Characterising heterogeneity | 21 | If applicable, report differences in costs, outcomes, or cost-effectiveness that can be explained by variations between subgroups of patients | Results; Discussion |
| ***Discussion*** |  |  |  |
| Findings, limitations, generalisability, and current knowledge | 22 | Summarize key findings and describe how they support the conclusions reached, and limitations to generalisability | Discussion |
| ***Other*** |  |  |  |
| Source of funding | 23 | Describe study funding and other non-monetary sources of support | Acknowledgements: |
| Conflicts of interest | 24 | Describe any potential conflicts of interest | Declaration of interests |

N/A: not available.

**Table S9. Testing volumes by intervention for cost proration.**

| **Implementation intervention** | **Estimated total monthly individuals tested at intervention cost level (for cost proration)** |
| --- | --- |
| Academic detailing (Lubelchek et al. [2013]) | Average of 16 tests per site per month (421 total tests/3 sites/9 months) |
| Educational support (Rhodes et al. [2016]) | Estimated 1,338 monthly individuals tested per health educator (based on citywide caseload of 100 clients per day and 21 working days per month) |
| All about me (Frye et al. [2020]) | Estimated 672 monthly individuals tested per recruiter (citywide based on 15 minutes per session, 8 hours per working day and 21 working days per month) |

**Table S10. Complete author responses to implementation intervention questionnaire.**

| **Lubelchek et al. (2013) - Academic detailing for HIV testing** Clinic-level costs and resource estimation | | | | | |
| --- | --- | --- | --- | --- | --- |
| There was no response provided by the author. Cost estimates are based on the source study and other sources found in Michaud et al. (2022).(10) | | | | | |
| *Intervention costing* | *Component of intervention* | *Staff type* | *Cost type* | *FTE or time commitment* | |
|  | Intervention administration (clerical, HR, etc.) | Admin | Implementation and sustainment (recurring monthly cost for full intervention period) | 0.25 FTE | |
|  | Develop training presentation | Program director | Implementation (one-time cost) | 6 hours | |
|  | Initial training presentation | Program director + all clinic staff | Implementation (one-time cost) | 1 hour | |
|  | Provider training | Physician | Implementation (one-time cost) | 1 hour | |
|  | Develop survey materials | Coordinator | Implementation (one-time cost) | 6 hours | |
|  | Feedback on survey from staff | All clinic staff | Implementation (one-time cost) | 0.25 hours | |
| **Rhodes et al. (2016) - CyBER/testing (HIV testing)** Jurisdiction-level costs and resource estimation (based on individuals tested per health educator) | | | | | |
| *Cost and resource requirement questions* | a.) How many individuals did a health educator need to talk to for every additional HIV test -- or how many health educator hours per additional HIV test taken?  **RESPONSE: Not available** b.) How many individuals could a health educator see in a day -- or what might a typical ‘caseload’ be? **RESPONSE: About 100** c.) How many admin or other support staff were required for the intervention -- or how many health educators could be supported by a particular number of admin staff?  **RESPONSE: 1 health educator; other staff detailed below.** | | | | |
| *Intervention costing* | *Component of intervention* | *Staff type* | *Cost type* | *FTE or time commitment* | *Additional costs* |
|  | Intervention administration (clerical, HR, etc.) – 0.2 FTE: 12,000/year + fringe, workers comp, payroll taxes | Admin – Executive director/supervision: 0.2 FTE: $18,000 + fringe, workers comp, payroll taxes | Implementation and sustainment (recurring monthly cost for full intervention period) | 1 FTE admin person per city | Local travel: $2000.00/year  4 community organizations (collaborators):  $10,000/year per organization  Space: $7,200/year  Telephone and internet: $2,400/year |
|  | Health educator – 1.0 FTE $36,000/year + fringe, workers comp, payroll taxes | Health educator | Delivery | 0.25 hours per individual regardless of whether visit results in test |  |
| **Frye et al. (2020) - All About Me (HIV testing)** Jurisdiction-level costs and resource estimation (based on individuals tested per recruiter) | | | | | |
| *Cost and resource requirement questions* | a.) How was information provided to participants? Was there active communication and recruitment, or was it passive (e.g., all advertising or web-based)?  **RESPONSE: We actively recruited participants using the methods described in the paper.**  b.) How many admin or other support staff were required for the intervention? How many admin or support staff would be required to operate the intervention at higher scales?  **RESPONSE: This data is impossible to estimate accurately in retrospect unfortunately. If I had to guess I would say one would need a 25% time outreach worker to identify and recruit men who could benefit from screening; this is a typical position in many CBOs. It could also just be deployed via social media platforms as an ad using the algorithm. If part of a CBO optimally there would also be a part-time case manager/navigator to monitor the app/survey/program and support users who may need personal support to get confirmatory testing or navigation to PrEP/PEP/ART etc. For receiving the self-test kits one would have to cover a supporting admin/organization staff member. And then there is the minimally supportive admin workers, like billing, payroll, reimbursement, grant writer for the funds from the state, etc.**  c.) Would development of a new web-based platform be necessary to scale up this intervention – or could it utilize existing platforms?  **RESPONSE: Creating the survey in a commercial platform would be a one-time cost.** | | | | |
| *Intervention costing* | *Component of intervention* | *Staff type* | *Cost type* | *FTE or time commitment* | |
|  | Intervention administration (clerical, HR, etc.) | Admin | Implementation and sustainment (recurring monthly cost for full intervention period) | 1 FTE admin person per city * **43.2%** [participants recommended to clinic-based testing] | |
|  | Develop survey and informational materials | Research coordinator | Implementation | 8 hours * **43.2%** [participants recommended to clinic-based testing] | |
|  | Identify and recruit potential testing candidates | Outreach worker/peer navigator | Delivery | 0.25 FTE outreach worker * **43.2%** [participants recommended to clinic-based testing] | |
|  | Online survey hosting | <Non-staff commitments> | Delivery | $250/month * **43.2%** | |
| **Storholm et al. (2021) - Project SLIP (PrEP)** Clinic-level costs and resource estimation | | | | | |
| There was no response provided by the author. Cost estimates are based on the source study and other sources found in Michaud et al. (2022).(10) | | | | | |
| *Intervention costing* | *Component of intervention* | *Staff type* | *Cost type* | *FTE or time commitment* | |
|  | Intervention administration (clerical, HR, etc.) | Admin | Implementation and sustainment (recurring monthly cost for full intervention period) | 0.25 FTE | |
|  | Develop training presentation | Program director | Implementation (one-time cost) | 6 hours | |
|  | Initial training presentation | Program director + all clinic staff | Implementation (one-time cost) | 1 hour | |
|  | Provider training | Physician | Implementation (one-time cost) | 1 hour | |
|  | Develop survey materials | Coordinator | Implementation (one-time cost) | 6 hours | |
|  | Feedback on survey from staff | All clinic staff | Implementation (one-time cost) | 0.25 hours | |
|  | Distribute and collect survey | Registered nurse | Delivery (recurring costs per individual initiating PrEP)* | 0.25 hours | |
| **Liu et al. (2019) – PrEPmate** Clinic-level costs and resource estimation | | | | | |
| *Cost and resource requirement questions* | a.) What percentage of ongoing clinic admin staff time would be devoted to the intervention?  **RESPONSE: Oak Tree women and family HIV clinic did a time-cost analysis in Vancouver (supporting HIV rx, not prep) was about 43min per patient per year of HCP time. Pediatric cardiology spends <1h per week to support 1000 patients. But you likely have PrEPmate experience with PCORI etc. Also to note, they found it time saving as texting was easier (less time) to follow than other means like phone/messages, outreach.**  b.) Are we accurately capturing the costs of setting up and maintaining a text messaging service for clinics? Would this be able to be integrated into existing appointment reminder services, or would it require more intensive setup? (our estimates were based on this source: <https://www.remindercall.com/blog/appointment-reminder-service-cost/>)  **RESPONSE: Not just a text reminder service - it is an interactive texting service as well (integrated patient engagement and virtual care)**  c.) Have we accurately captured implementation and setup costs that might be incurred by a typical clinic?  **RESPONSE: N/A**  d.) Do you agree with the assumption of no delivery costs for the intervention (i.e., text messages to individuals would be the only incremental ‘cost’ for PrEP patients, which we assumed to be negligible)?  **RESPONSE: Receiving texts is free to patients depending on the plan, there may be small costs sending messages in, but those are often offset by saving time and money traveling. Since patients can get advice etc. via PrEPmate on their phones, it actually saves them substantial time vs coming to clinic or phoning the clinic. (time/cost savings on patient from virtual care)** | | | | |
| *Intervention costing* | *Component of intervention* | *Staff type* | *Cost type* | *FTE or time commitment* | |
|  | Intervention administration (clerical, HR, etc.) | Navigator | Implementation and sustainment (recurring monthly cost for full intervention period) | 0.05 FTE (based on number of clients enrolled) | |
|  | Text messaging setup | IT (or external vendor) | Implementation (one-time cost) | $1000 per clinic | |
|  | Develop training presentation | Program director | Implementation (one-time cost) | 1 hour | |
|  | Training | Program director + all clinic staff | Implementation (one-time cost) | 1 hour / $1000 | |
|  | Subscription and maintenance of text reminder | IT (or external vendor) | Sustainment (monthly) | $75 per patient per year | |
| **Doblecki-Lewis et al. (2019) - PrEP patient navigation** Clinic-level costs and resource estimation | | | | | |
| *Cost and resource requirement questions* | a.) What percentage of ongoing clinic admin staff time would be devoted to the intervention (we are currently assuming 0.25 FTE for one admin)?  **RESPONSE: Agree**  b.) Have we attributed enough time to patient navigator training?  **RESPONSE: Our navigators received 2 x 2 hour sessions (total 4 hours) on strengths-based navigation and motivational interviewing, including role play, resource review, and troubleshooting. I think 1 hour is too little, probably 2.5 hours would be a minimum.**  c.) What would a typical patient navigator caseload be (or how many patient navigators per clinic)?  **RESPONSE: Typical patient navigator caseload is about 75 active cases this is for both initial navigation and follow-up.** | | | | |
| *Intervention costing* | *Component of intervention* | *Staff type* | *Cost type* | *FTE or time commitment* | |
|  | Intervention administration (clerical, HR, etc.) | Admin | Implementation and sustainment (recurring monthly cost for full intervention period) | 0.25 FTE | |
|  | Patient navigator training | Patient navigator + trainer/program director | Implementation (one-time cost) | 4 hour per patient navigator and trainer | |
|  | Patient navigator visit | Patient navigator | Delivery (per person) | 2 hours per person* [median 2 (1-5) visits; initial visit + follow-up] | |
|  | Space | <Non-staff commitments> | Delivery | $7,200/year; $600/month | |
|  | Cellular phone for patient navigators | <Non-staff commitments> | Delivery | $100/month per patient navigator | |

Authors of the source studies used in this analysis were contacted by the research team to provide further details related to costs and population-level scaling factors of their respective interventions. Initial contact was made with the corresponding author, and additional study authors were contacted at the discretion of the corresponding author. Any additional information provided by the source study author(s) was included in the model’s estimates for population-level scaling factors or intervention costs.
HIV – human immunodeficiency virus; FTE – full-time equivalent; HR – human resources; CyBER: Cyber-Based Education and Referral; CBO – community-based organization; PrEP – pre-exposure prophylaxis; PEP - Post-exposure prophylaxis; ART - Antiretroviral therapy; SLIP: Screening and Linkage Intervention in Primary Care; HCP – health care provider; PCORI – patient-centered outcomes research institute.

**Figure S1. Dynamic compartmental HIV model diagram**

**
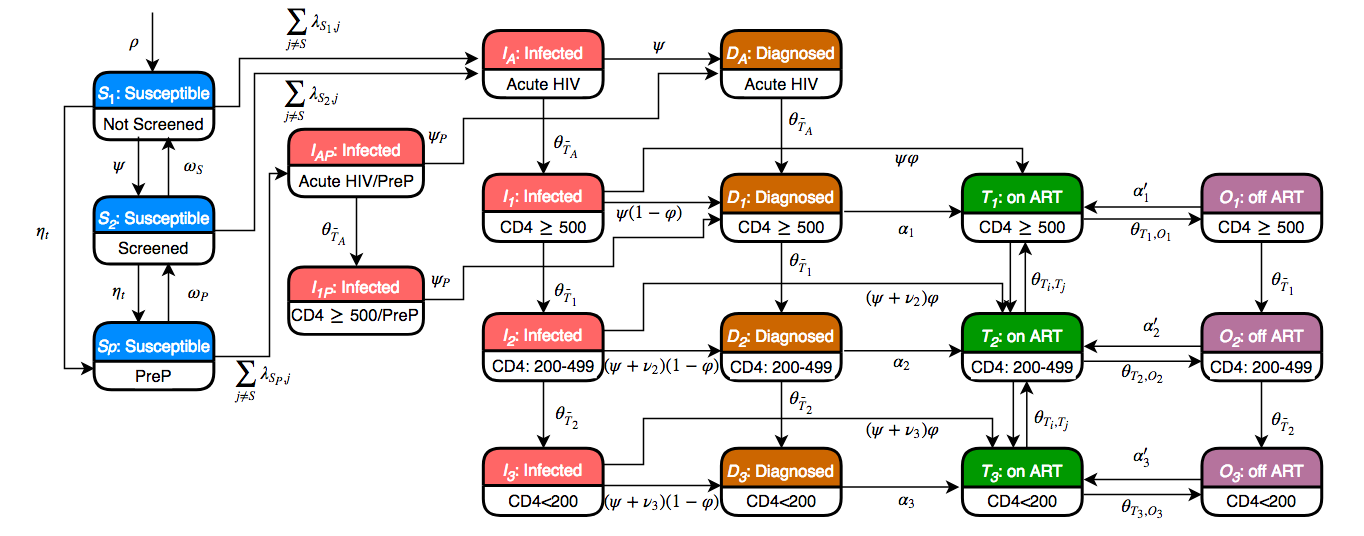
**
